# Supplementary material for: Separating natural from human enhanced methane emissions in headwater streams
Source: Nat Commun. 2022 Jul 1;13:3810. doi: 10.1038/s41467-022-31559-y (PMC9249869; doi:10.1038/s41467-022-31559-y)
Supplement: Supplementary file 1 — Supplementary Information [file 41467_2022_31559_MOESM1_ESM.pdf]

Supplementary Information

Separating natural from human enhanced methane emissions in headwater streams

Authors: Yizhu Zhu[1], J. Iwan Jones[1], Adrian L. Collins[2], Yusheng Zhang[2], Louise Olde[1,2], Lorenzo Rovelli[3], John F. Murphy[1], Catherine M. Heppell[4], Mark Trimmer[1]*

Affiliations:

[1]School of Biological and Behavioural Sciences, Queen Mary University of London, London, E1 4NS, UK.

[2]Sustainable Agriculture Sciences, Rothamsted Research, North Wyke, Okehampton, Devon, EX20 2SB, UK.

[3]Institute for Environmental Sciences, University of Koblenz-Landau, Landau, Germany

[4]School of Geography, Queen Mary University of London, London, E1 4NS, UK.

*Correspondence to: Mark Trimmer m.trimmer@qmul.ac.uk

Supplementary Figure 1 | Positions of the streams sampled in southern UK. The white dots are the 14 streams where sediments were collected for our laboratory incubations (see also Supplementary Table 1). The coloured dots are the 29 streams where water samples were collected to estimate methane emissions in relation to the sediment pressure categories described in the main text. The map was created using the map_data() function in the R package ggplot2 (version 3.0.0)[1].

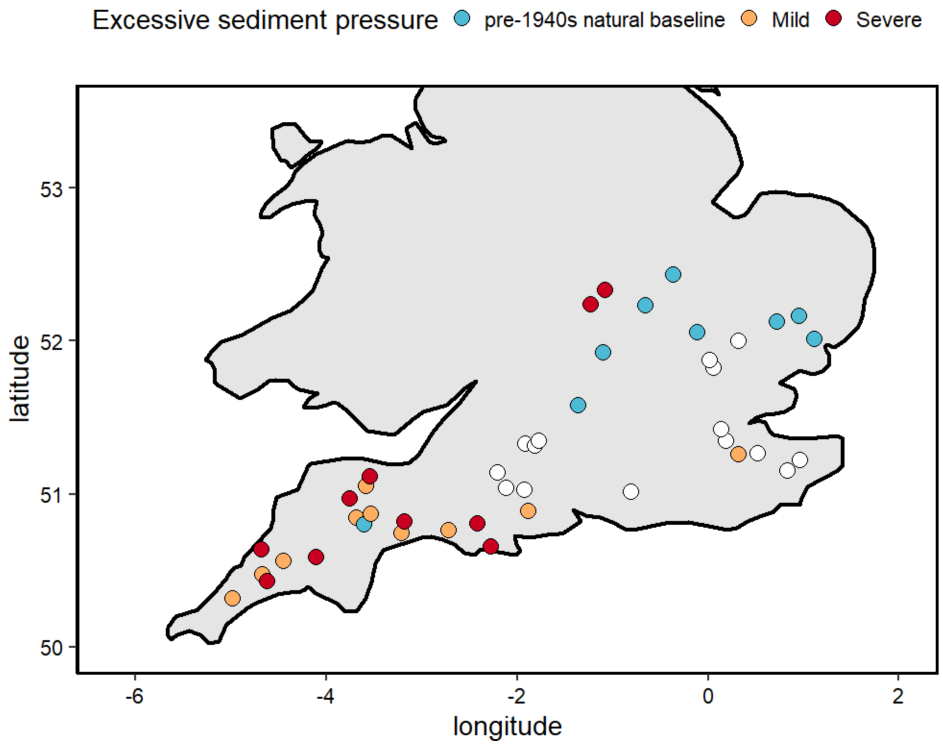

Supplementary Figure 2 | Methane production capacity of three sediment patch types (*n*=371, streams=14). Patches of aquatic plants which trap fine sediments, rich in organic matter, create hotspots of methane production[2] on streambeds. These plant patch sediments contained the highest organic matter at 1,715 g m^{-2} and had the strongest methane production at 1.58 nmol CH_4 g^{-1} h^{-1}. Stream marginal patches contained 1,435 g m^{-2} organic matter and produced 0.13 nmol CH_4 g^{-1} h^{-1}, while main channel sediments contained 1,019 g m^{-2} organic matter and produced 0.09 nmol CH_4 g^{-1} h^{-1}. Overall, both the marginal and main-channel sediments produced less methane than sediment deposited under the plant patches (*post-hoc* analysis for pairwise comparison using Tukey method, $P<0.001$, see Supplementary Table 4), strongly suggesting methane production capacity was related to sediment organic matter content. The organic matter on streambed was measured as ash-free-dry-weight in unit of g per m^2. The filled circles represent the capacity of methane production at 15°C estimated for each patch type using a linear mixed-effect model (see Supplementary Table 4) and the vertical lines are 95% CI.

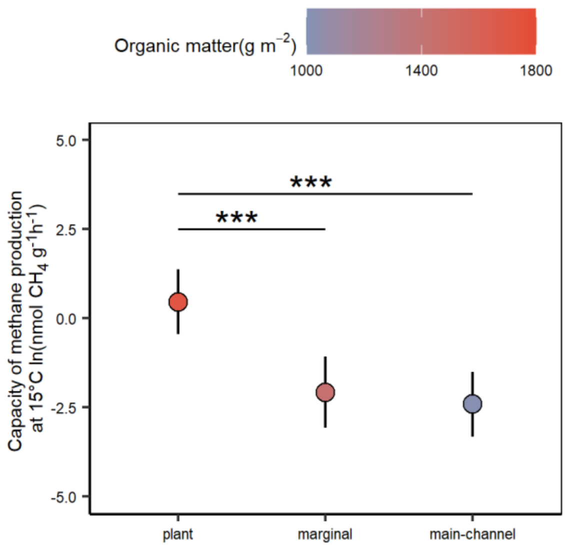

Supplementary Figure 3 | Literature derived methane production in river sediments[3–5] (*n*=58), lake sediments[6–10] (*n*=78) and wetland soils[11–14] (*n*=18) in relation to their organic matter content. a, The increase in methane production per 10-fold rise in sediment organic matter content, i.e., the slopes, was comparably high in both river and wetlands, but lower in lakes (***: P<0.001, two-sided likelihood ratio test). **b**, Despite the clear distinction between the three habitat types in their respective fold increase in methane production per 10-fold rise in organic matter content (i.e., the different slopes in panel **a,** here extracted and presented on the y-axis), there is no relationship between the slopes and the average incubation temperature used for each habitat type. The unit of organic matter content is % ash-free-dry-weight. Vertical lines in **b** are 95% CI. A linear-mixed-effect model was used to characterize the overall increase in rate of methane production in relation to organic matter between habitat types (coloured solid lines in panel **a**). The variation between studies, including incubation temperature mentioned above, was accounted for by including each study site as a random effect.

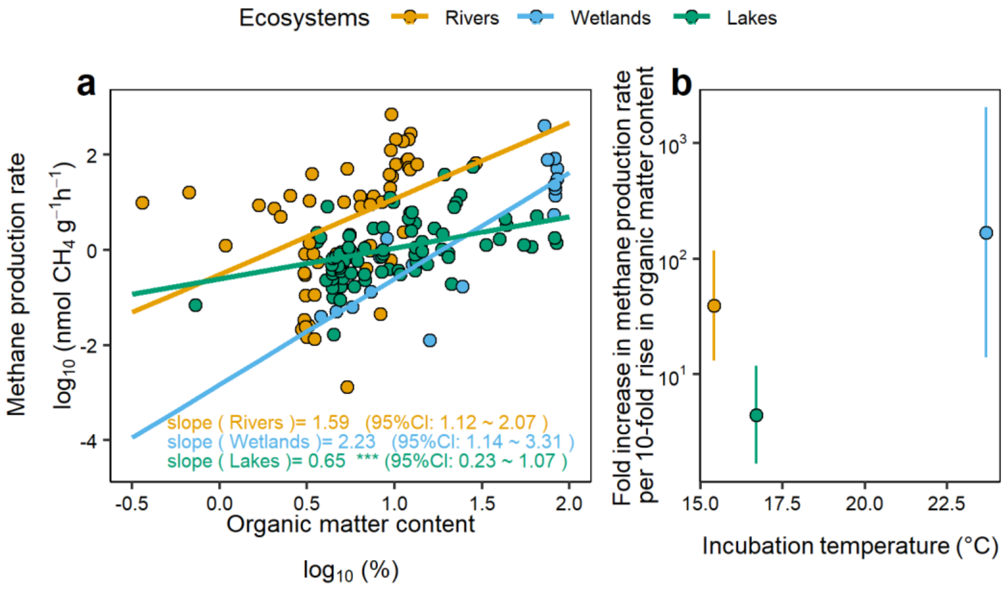

Supplementary Figure 4 | River median flow (Q_{50}) as a function of median annual maximum flow (Q_{MED}) in England and Wales. Note, out of 704 observations provided by the National River Flow Archive, 536 had Q_{MED} values in the same range as our 236 study streams ($0<Q_{MED}<82$ m^3 s^{-1}) and we used those for our extrapolation. The black solid line represents the median regression fitted to the data ("quantreg" package 5.86)[15] and the slope of 0.034 was used in the main text to estimate Q_{50} based on the available Q_{MED} data.

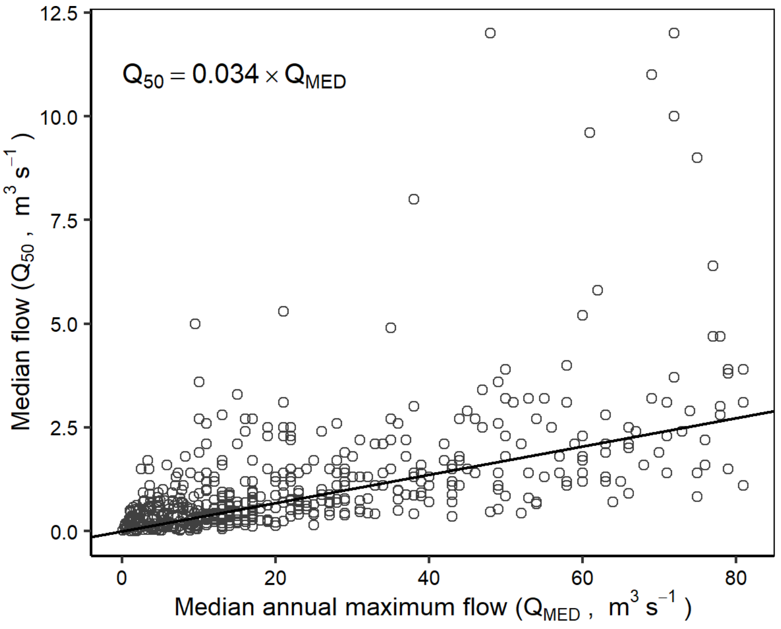

Supplementary Table 1 | Statistical summary of geomorphological and hydrological data for the 236 study streams. Q1 and Q3 represent the first and third quartiles, respectively, for the dataset.

	Mean (±SE)	Min	Q1	Median	Q3	Max
Catchment area (km^2)	11.2 (±0.84)	0.52	2.6	7.5	15.4	114.2
Distance from source (km)	5.1 (±0.26)	0.41	2.1	4.4	6.6	31.4
Elevation above sea-level (m)	131.6 (±5.6)	4	60	120	188.2	467.2
Slope (m km^{-1})	18.8 (±1.6)	0.4	5.2	10	23.4	214.1
Water width (m)	3.5 (±0.16)	0.6	1.9	2.9	4.5	14.5
Median annual maximum flow (Q_{MED}, $m^3 s^{-1}$)	8.8 (±0.7)	0.02	1.5	4.6	13	82
Median flow (Q_{50}, $m^3 s^{-1}$)	0.2 (±0.02)	<0.001	<0.001	0.1	0.3	2.8

Supplementary Table 2 | Sampling date and sediment particle size for the subset of streams used in the incubations. Sediment particle size was classified on the Wentworth scale as: silts (<62.5μm), sands (62.5–2000μm), gravels (2000–4000μm) and pebbles (>4000μm)[16] and sediments were predominantly either sands or gravels. Sediment particle size had a similar distribution between streams on either predominant geology i.e., Chalk or Greensand and streams are ranked by increasing particle size within each dominant geology. Geology type was based on British Geological Survey data or related research papers[17–19].

Stream	Geology	Patch	Sampling date	Particle sizes (Median diameter, μm)	Sediment type
Wylye	Chalk	plant	February 2013	29	silt
Wylye	Chalk	marginal	February 2013	148	sand
Rushall-Chalk	Chalk	main	August 2013	167	sand
Wylye	Chalk	marginal	August 2013	211	sand
Rushall-Chalk	Chalk	main	February 2013	274	sand
Stour2	Chalk	plant	September 2016	325	sand
Rib	Chalk	plant	February 2016	329	sand
Stour1	Chalk	plant	September 2016	331	sand
Darent	Chalk	plant	March 2016	353	sand
Ash	Chalk	plant	March 2016	807	sand
Ebble	Chalk	main	August 2013	1600	sand
Pant	Chalk	plant	May 2016	2124	gravel
Wylye	Chalk	plant	August 2013	3375	gravel
Wylye	Chalk	main	August 2013	3875	gravel
Ebble	Chalk	main	February 2013	4950	pebble
Wylye	Chalk	main	February 2013	8200	pebble
Marden-Avon	Greensand	main	February 2013	88	sand
Marden-Avon	Greensand	marginal	February 2013	196	sand
Marden-Avon	Greensand	marginal	August 2013	207	sand
Marden-Avon	Greensand	main	August 2013	298	sand
Medway	Greensand	plant	June 2016	324	sand
Hammer Stream	Greensand	plant	April 2016	382	sand
Nadder	Greensand	main	August 2013	419	sand
Marden-Avon	Greensand	plant	August 2013	530	sand
Nadder	Greensand	main	February 2013	904	sand
Rushall-Sand	Greensand	main	August 2013	3650	gravel

Supplementary Table 3 | Model selection procedure for fitting linear models to the streambed organic matter data (*n*=236). We used a backward stepwise selection procedure to select the best fitting model to streambed organic matter data. The full model included additive terms and their interaction for two predictors – $\log_{10}$ scale of standardized delivery of excess fine sediments ($\log_{10}$SD, see equation (7) of main text) and excess fine sediment pressure category (Category, i.e., pre-1940s natural baseline, mild or severe). The significance of each term was determined via a two-sided *F*-test comparing the nested models and non-significant terms were eliminated stepwise. Model F1, marked in bold, provided the best fit to the streambed organic matter data and also had the lowest AIC value. Model F1 included a single slope but different intercepts across the sediment pressure categories, demonstrating that organic matter deposited on streambeds differed across sediment pressure categories but increased in parallel with standardized delivery of excess fine sediments to streams. To visualise this main effect, we created partial residual plots using the R package visreg[20] e.g. in Figure 1b we illustrate the overall relationship between streambed organic matter and standardized excessive fine sediment delivery in the best-fitting model (here Model F1) while holding excessive sediment pressure constant at its median value.

Model	AIC	*F*	*P*
F0) $\log_{10}$OM~$\log_{10}$SD*Category	352.5		
**F1) $\log_{10}$OM~$\log_{10}$SD+Category**	**349.9**	**0.67**	**0.51**
F2) $\log_{10}$OM~$\log_{10}$SD	416.4	40.4	<0.001
F3) $\log_{10}$OM~Category	388.0	43.1	<0.001

Supplementary Table 4 | Model selection procedures for fitting linear mixed-effect models to the rate of methane production in laboratory incubations (*n*=371, streams=14). The full model included additive terms and their interactions for 2 fixed effects – standardized temperature (Ts, the term $(\frac{1}{kT_{15}} - \frac{1}{kT_{\mathrm{ij}}})$ in equation (8) of the main text) and patch type (i.e., plant, marginal and main-channel). Random-effects were first included on both the intercept and the slope to account for any variation among sampling dates within streams (1+Ts|Stream:Month) (see Supplementary Table 1 for the list of streams). The significance of random effects was tested using a two-sided likelihood ratio test by comparing the full and reduced models and non-significant terms were eliminated stepwise. The random-intercept-only structure i.e., variation among sampling dates within streams appeared to be the preferred option (marked in bold) suggesting a conserved temperature sensitivity across streams and was applied for subsequent significance testing of fixed-effects. *P*-values for fixed-effect terms were determined via two-sided likelihood ratio testing. Model F1 (marked in bold) provided the best fit to the methane production data with the lowest AIC value. Model F1 included a single slope but different intercepts across patch types, demonstrating that while streambed sediments had different methane production capacities across patch types at a standardized 15°C, the temperature sensitivity of that methane production was conserved. As we focused on the capacity of methane production in each stream and its correlation to sediment organic matter, the effect of patch type is not presented in the main text but in Supplementary Figure 2.

Model	d.f.	AIC	LogLik	χ^2	*P*
To determine the optimal random-effects structure:					
R0) lnMG~Ts*Patch+(1+Ts|Stream:Month)	10	1586.4	-783.19		
**R1) lnMG~Ts*Patch+(1|Stream:Month)**	**8**	**1583.5**	**-783.75**	**0.57**	
R2) lnMG~Ts*Patch	7	1774.5	-880.25	192.9	<0.001
To determine the optimal fixed-effects structure:					
F0) lnMG~Ts*Patch+(1|Stream: Month)	8	1583.5	-783.75		
**F1) lnMG~Ts+Patch+(1|Stream: Month)**	**6**	**1580.2**	**-784.08**	**0.65**	**0.72**
F2) lnMG~Ts+(1|Stream: Month)	4	1643.6	-817.79	67.43	<0.001
F3) lnMG~Patch+(1|Stream: Month)	5	1673.7	-831.86	95.57	<0.001

Supplementary Table 5 | Model selection procedures for fitting linear mixed-effects models to methane production as a function of temperature and additional substrates (*n*=571, streams=8). The full model included additive terms and their interactions for 2 fixed effects – standardized temperature (Ts, term $(\frac{1}{kT_{15}} - \frac{1}{kT_{ij}})$, see equation (10) in the main text) and additional substrates (see main text for a full description). Random effects (1+Ts|Streams) were first included on both the intercept and the slope to account for any variation between streams (see Supplementary Table S1 for the list of streams), their significance determined using a two-sided likelihood ratio test and any non-significant terms were removed stepwise. For example, despite models R0 and R1 having the same AIC value, inclusion of a random-slope effect did not improve the model fit (*P*-value = 0.13) and was thus removed. The random intercept only model appeared to be the preferred option (R1, marked in bold) and was applied for subsequent testing of the fixed effects, where *P*-values for significant fixed-effect terms were determined via two-sided likelihood ratio testing. After removing the non-significant interaction term Ts:Substrate, Model F1 provided the best fit to the methane production data (F1, marked in bold). Model F1 included a single temperature sensitivity term but separate intercepts among the additional substrates to produce methane, suggesting that while the addition of substrates increased the methane production capacity of the sediments, the temperature sensitivity was conserved.

**Model**	**d.f.**	**AIC**	**LogLik**	χ^2	***P***
To determine the optimal random-effects structure:					
R0) lnMG~Ts*Substrates+(1+Ts|Streams)	16	1758.9	-863.45		
**R1) lnMG~Ts*Substrates +(1|Streams)**	**14**	**1758.9**	**-865.46**	**4.01**	**0.13**
R2) lnMG~Ts*Substrates	13	2112.4	-1043.22	355.52	<0.001
To determine the optimal fixed-effects structure:					
F0) lnMG~Ts*Substrates	14	1758.9	-865.46		
**F1) lnMG~Ts+Substrates**	**9**	**1756.5**	**-869.23**	**7.54**	**0.18**
F2) lnMG~ Substrates	8	1943.5	-963.73	189.01	<0.001
F3) lnMG~Ts	4	1977.2	-869.23	230.73	<0.001

Supplementary Table 6 | Model selection procedures for fitting linear mixed-effects models to the standardized methane emissions ($\log_{10}$SME) as a function of standardized delivery of excess fine sediments (*n*=142, streams=29). The full model included additive terms and their interactions for 2 fixed effects – the $\log_{10}$ scale of standardized delivery of excess fine sediments ($\log_{10}$SD, see equation (13) of main text) and excess fine sediment pressure categories, i.e., pre-1940s natural baseline, mild or severe. As five replicates were collected to estimate methane emissions from each stream but the delivery of excess fine sediments were estimated only once, random effects (1|Streams) were included on the intercept only to account for the variation in standardized emissions of methane within each stream. The significance of the fixed effects terms were determined using a two-sided likelihood ratio test. Model F1 (marked in bold) provided the best fit to the standardized methane emission data and included a single slope i.e., standardized methane emissions increased in parallel with standardized delivery of excess fine sediment. To facilitate visualization, partial residual plots were created using the R package visreg[20] where, in Figure 4a, we illustrate the relationship between standardized methane emission and standardized excessive fine sediment delivery in the best-fitting model (here the Model F1) while holding the other variables constant (here excessive sediment pressure at its median value).

**Model**	**d.f.**	**AIC**	**LogLik**	χ^2	***P***
The full model with its fixed and random effects:					
R0) $\log_{10}$SME~$\log_{10}$SD*Category+(1|Streams)					
To determine the optimal fixed-effects structure:					
F0) $\log_{10}$SME~$\log_{10}$SD*Category+(1|Streams)	8	-189.88	102.94		
**F1) $\log_{10}$SME~$\log_{10}$SD+Category+(1|Streams)**	**6**	**-192.84**	**102.42**	**1.04**	**0.59**
F2) $\log_{10}$SME~$\log_{10}$SD+(1|Streams)	4	-181.97	94.99	14.87	<0.001
F3) $\log_{10}$SME~Category+(1|Streams)	5	-188.31	99.16	6.52	<0.05

Supplementary references

1. Wickham, H. *ggplot2: Elegant Graphics for Data Analysis*. (Springer-Verlag New York, 2016).
2. Sanders, I. A. *et al.* Emission of methane from chalk streams has potential implications for agricultural practices. *Freshw. Biol.* **52**, 1176–1186 (2007).
3. Crawford, J. T. & Stanley, E. H. Controls on methane concentrations and fluxes in streams draining human-dominated landscapes. *Ecol. Appl.* **26**, 1581–1591 (2016).
4. Mach, V., Blaser, M. B., Claus, P., Chaudhary, P. P. & Rulík, M. Methane production potentials, pathways, and communities of methanogens in vertical sediment profiles of river Sitka. *Front. Microbiol.* **6**, 1–12 (2015).
5. Shelley, F., Abdullahi, F., Grey, J. & Trimmer, M. Microbial methane cycling in the bed of a chalk river: oxidation has the potential to match methanogenesis enhanced by warming. *Freshw. Biol.* **60**, 150–160 (2015).
6. Conrad, R. *et al.* Stable carbon isotope discrimination and microbiology of methane formation in tropical anoxic lake sediments. *Biogeosciences* **8**, 795–814 (2011).
7. Li, B. *et al.* Methane distribution patterns along a transect of Lake Fuxian, a deep oligotrophic lake in China. *Environ. Sci. Pollut. Res.* **27**, 25848–25860 (2020).
8. Karvinen, A., Lehtinen, L. & Kankaala, P. Variable Effects of Iron (Fe (III)) Additions on Potential Methane Production in Boreal Lake Littoral Sediments. *Wetlands* **35**, 137–146 (2014).
9. Nozhevnikova, A. N., Zepp, K., Vazquez, F., Zehnder, A. J. B. & Holliger, C. Evidence for the existence of psychrophilic methanogenic communities in anoxic sediments of deep lakes. *Appl. Environ. Microbiol.* **69**, 1832–1835 (2003).
10. Duc, N. T., Crill, P. & Bastviken, D. Implications of temperature and sediment characteristics on methane formation and oxidation in lake sediments. *Biogeochemistry* **100**, 185–196 (2010).
11. Inglett, K. S., Inglett, P. W., Reddy, K. R. & Osborne, T. Z. Temperature sensitivity of greenhouse gas production in wetland soils of different vegetation. *Biogeochemistry* **108**, 77–90 (2012).
12. Liu, D. Y., Ding, W. X., Jia, Z. J. & Cai, Z. C. Relation between methanogenic archaea and methane production potential in selected natural wetland ecosystems across China. *Biogeosciences* **8**, 329–338 (2011).
13. Valentine, D. W., Holland, E. A. & Schimel, D. S. Ecosystem and physiological controls over methane production in northern wetlands. *J. Geophys. Res. Atmos.* **99**, 1563–1571

(1994).

14. Wright, A. L. & Reddy, K. R. Heterotrophic Microbial Activity in Northern Everglades Wetland Soils. *Soil Sci. Soc. Am. J.* **65**, 1856–1864 (2001).

15. Koenker, R. quantreg: Quantile Regression. (2021).

16. Wentworth, C. K. A Scale of Grade and Class Terms for Clastic Sediments. *J. Geol.* **30**, 377–392 (1922).

17. Heppell, C. M. *et al.* Hydrological controls on DOC nitrate resource stoichiometry in a lowland, agricultural catchment, southern UK. *Hydrol. Earth Syst. Sci.* **21**, 4785–4802 (2017).

18. Bridgland, D. R. The evolution of the river Medway, SE England, in the context of Quaternary palaeoclimate and the palaeolithic occupation of NW Europe. *Proc. Geol. Assoc.* **114**, 23–48 (2003).

19. Foster, I. D. L. *et al.* Anthropogenic sediment traps and network dislocation in a lowland UK river. *Earth Surf. Process. Landforms* 1–16 (2021) doi:10.1002/esp.5235.

20. Breheny, P. & Burchett, W. Visualization of Regression Models Using visreg. *R J.* **9**, 56–71 (2017).
