# Supplementary material for: Separating natural from human enhanced methane emissions in headwater streams
Source: Nat Commun. 2022 Jul 1;13:3810. doi: 10.1038/s41467-022-31559-y (PMC9249869; doi:10.1038/s41467-022-31559-y)
Supplement: Supplementary file 3 — Reporting Summary [file 41467_2022_31559_MOESM3_ESM.pdf]

nature research

Corresponding author(s): Mark Trimmer

Last updated by author(s): Jun 8, 2022

Reporting Summary

Nature Research wishes to improve the reproducibility of the work that we publish. This form provides structure for consistency and transparency in reporting. For further information on Nature Research policies, see our Editorial Policies and the Editorial Policy Checklist.

Statistics

For all statistical analyses, confirm that the following items are present in the figure legend, table legend, main text, or Methods section.

n/a	Confirmed	
☐	☒	The exact sample size (*n*) for each experimental group/condition, given as a discrete number and unit of measurement
☐	☒	A statement on whether measurements were taken from distinct samples or whether the same sample was measured repeatedly
☐	☒	The statistical test(s) used AND whether they are one- or two-sided<br>*Only common tests should be described solely by name; describe more complex techniques in the Methods section.*
☐	☒	A description of all covariates tested
☒	☐	A description of any assumptions or corrections, such as tests of normality and adjustment for multiple comparisons
☐	☒	A full description of the statistical parameters including central tendency (e.g. means) or other basic estimates (e.g. regression coefficient) AND variation (e.g. standard deviation) or associated estimates of uncertainty (e.g. confidence intervals)
☐	☒	For null hypothesis testing, the test statistic (e.g. *F*, *t*, *r*) with confidence intervals, effect sizes, degrees of freedom and *P* value noted<br>*Give P values as exact values whenever suitable.*
☒	☐	For Bayesian analysis, information on the choice of priors and Markov chain Monte Carlo settings
☒	☐	For hierarchical and complex designs, identification of the appropriate level for tests and full reporting of outcomes
☒	☐	Estimates of effect sizes (e.g. Cohen's *d*, Pearson's *r*), indicating how they were calculated

Our web collection on statistics for biologists contains articles on many of the points above.

Software and code

Policy information about availability of computer code

Data collection	"GC ChemStation Software rev A.10.02" was used for gas chromatogram data collection.
Data analysis	Mixed-effect models were fitted and analysed using lme4' package (version 1.1-23) and all statistical analyses were performed in R software (version 4.0.0).

For manuscripts utilizing custom algorithms or software that are central to the research but not yet described in published literature, software must be made available to editors and reviewers. We strongly encourage code deposition in a community repository (e.g. GitHub). See the Nature Research guidelines for submitting code & software for further information.

Data

Policy information about availability of data

All manuscripts must include a data availability statement. This statement should provide the following information, where applicable:

- Accession codes, unique identifiers, or web links for publicly available datasets
- A list of figures that have associated raw data
- A description of any restrictions on data availability

Data generated in this study (including the raw data associated with Figures 1 to 4 in the main text) are provided in the Source Data file.

Field-specific reporting

Please select the one below that is the best fit for your research. If you are not sure, read the appropriate sections before making your selection.

☐ Life sciences ☐ Behavioural & social sciences ☒ Ecological, evolutionary & environmental sciences

For a reference copy of the document with all sections, see nature.com/documents/nr-reporting-summary-flat.pdf

Ecological, evolutionary & environmental sciences study design

All studies must disclose on these points even when the disclosure is negative.

Study description	Several studies have previously reported correlations between streambed organic matter and stream water methane, along with higher sediment rates of methane production and it is also recognized that streams in agricultural catchments can also be associated with higher methane. However, there has been no systematic assessment of the widespread ingress of agriculturally derived fine sediment, how that affects streambed organic matter and, in turn, methane production or how the magnitude of that effect compares to one induced by climate warming to date. We first demonstrate for 236 UK streams that excess fine sediment is widespread and since the 1940s has increased streambed organic matter from 23 to 100 g AFDW per m2. Through laboratory incubations of fine sediment for 14 other streams, we show that the response of streambed methane production to temperature is consistent but that the increase in production in relation to organic matter is up to ten-thousand fold. The increase in methane production was further vindicated by the fact that methane emissions from a subset of 29 streams selected from the 236 excess fine sediment database, have indeed tripled from 0.2 to 0.7 mmol CH4 per m2 per d with excess fine sediment delivery against the same pre-1940s natural baseline. While streambed methane production responds strongly to organic matter, we estimate the effect of warming since the 1940s (~0.7°C) to be comparatively modest. This study separates natural from human enhanced methane emissions and highlights how catchment management targeting the delivery of excess fine sediment could mitigate stream methane emissions.
Research sample	Of the 236 study streams, 182 were selected from the 12,447 sites within the Environment Agency River Habitat Survey (RHS) database with any sites influenced by urban areas or sewage effluent eliminated via screening and the other 54 were selected according to the extent of participation in agri-environment schemes in their catchments. The fine sediment delivery was computed using the PSYCHIC model with corrections for the impacts of the current uptake of on-farm best practice. The fine sediment samples for streambed organic matter measurement were collected between May 2010 and November 2011 using the disturbance technique as previously published (see ref27 in the main text). To characterize the reach-scale average, four locations (2 erosional and 2 depositional) were sampled from each stream. The subsequent sediment samples used for laboratory incubations were collected in 2013 and 2016 from streambeds in streams in two dominant geologies (Chalk and Greensand/sand) including three different patch types (fine sediments underneath vegetation, marginal and main-channel). The choice of the 14 streams was based on field work experience and site accessibility. The sediments were collected using corers and the sub-samples of the bottom 3 to 5 cm of sediments were used for incubation because the methanogens are active in the deeper, anoxic layers. For the final methane emission estimates, water samples were collected from the middle of the stream channel in August 2020 from a subset of 29 streams selected from the 236 study sites according to field trip availability but the number of streams were approximately equal for each excess sediment pressure category, i.e., pre-1940s natural baseline (n=9, as one sample was lost), mild (n=10) or severe (n=10) to avoid any bias.
Sampling strategy	We did not perform any prior power analysis to determine the minimum sample size. However, the organic matter on the 236 streambeds with available excess fine sediment delivery data provided by co-authors A.L.C., Y.-S.Z., J.I.J. and J.F.M. are the maximum number of streams we could include in this study. Furthermore, our previous work in the similar UK streams (see Shelley et al. ref44 and Sanders et al. ref19) - despite of their relatively small sampling site numbers - have demonstrated good precision in potential and temperature sensitivity of methane production. Here, we combined the data collected by two co-authors L.O. and Y.Z. from their field trips collecting sediments from 14 independent streams, collecting between 3 to 4 "technical-replicates" within each stream that were used for the subsequent laboratory incubations. By doing so, we gained strong statistical power to prove a consistent temperature sensitivity of methane production across all 14 streams, despite the large variation in their capacity to produce methane and noting that the statistical models are penalised for the repeat measures in any one stream (see below). For methane emissions, water samples were collected in August 2020 and the number of streams for sampling was restricted to 30, with 5 "technical-replicates" within each stream, due to the COVID travel restrictions in the UK, but the number of streams were approximately equal for each excess sediment pressure category, i.e., pre-1940s natural baseline(n=9, as one sample was lost), mild (n=10) or severe (n=10) to avoid any bias.
Data collection	The estimates of excess fine sediment delivery were computed using the PSYCHIC model by A.L.C. and Y.-S.Z with corrections for the impacts of the current uptake of on-farm best practice for water quality protection driven by regulation, incentivisation and advice - that is published and described extensively elsewhere. Streambed organic matter was quantified using the disturbance technique from four locations (2 erosional and 2 depositional) in each stream by J.I.J. and J.F.M as described elsewhere and in the main text here. Streambed sediments, with 3 to 4 "technical-replicates" for each stream, were collected using sediment corers by Y.Z. and L.O. For estimating methane emissions, water samples were collected from the middle of stream main channels using polytetrafluoroethylene tubing attached to a 60 ml gas-tight syringe by Y.Z. and J.I.J. Then, in the laboratory, methane concentrations and methane production rates in incubations were measured by injecting 100 µl of headspace into a gas chromatograph fitted with a flame-ionizing detector and data were recorded by "GC ChemStation Software" then collected by Y.Z. and L.O.
Timing and spatial scale	The fine sediment samples for organic matter content measurement were collected between May 2010 and November 2011 and the positions of the 236 streams are visualized in Figure 1a of the main text. In 2013, streambed sediments were collected from 6 streams in February and August. In 2016, further streambed sediments were collected from another 8 streams but once only through out February to September (see Supplementary Table 2 for more details). Each field trip was restricted to one day. The streams are in

the south of England, UK and their positions are provided in Supplementary Figure 1. For emissions, water samples were collected in a subset of 29 streams of the total 236 streams in August 2020 and their positions are provided in Supplementary Figure 1.
For laboratory incubations, each vial containing streambed sediments (as above) were incubated for up to 4 days during which period the concentrations of methane in the headspace was measured once each day.

Data exclusions

Of a total of 371 sediment samples incubated throughout the study, 3 were excluded from any statistical analysis due to having no detectable methane production during incubations. A further 3 water samples used to estimate methane emissions were also excluded due to no detectable methane, probably, in both cases, due to a analytical error e.g. a blocked needle on the GC.

Reproducibility

In this study, we collected sediment samples from 14 streams on two dominant geologies (Chalk, typically with gravel beds and Greensand or sand, with typically sand beds) to provide good representation of the dominant UK streambed types. For 6 of the 14 streams, each stream was visited twice, once in February, 2013, and in August, 2013, (i.e., winter and summer) and the sediments from three patch types (fine sediments underneath vegetation, marginal and main-channel) were collected. For the other 8 of the 14 streams, each stream was visited only once in 2016 and the sediments were collected from the vegetated patches, only. Moreover, in each of the 14 streams, three to four "technical-replicates" of sediments were collected per visit per patch type. To investigate the temperature sensitivity and the rate of methane production standardized to the same 15 °C, rates of methane production were recorded from further sub-samples of sediment incubated under a temperature-gradient from 5°C to 26°C and were fitted into one mixed-effects model using each stream and their sampling date as random effects. Despite the large variation in their capacity to produce methane, the 14 streams, and their "technical-replicates" within each stream, therefore granted us a strong capacity not only to account for the variation within each stream but also to estimate an overall temperature sensitivity of methane production across all 14 streams. Similarly, for the later sediment samples collected from 8 streams in 2016 to test the effect of additional substrates, the 3 to 4 "technical-replicates" within each stream were fitted into a mixed-effects model using each stream as a random effect. These additional substrate incubations once again demonstrated a consistent temperature sensitivity of methane production despite of the stimulation in methane production caused by the addition of substrates.

Randomization

All of the sediment core samples collected from streambeds were treated the same. After transferring sediments into gas-tight vials for incubations, the vials were randomly allocated for additional substrates and incubation to allow randomization.

Blinding

The incubation data reported in this study form part of the PhD theses for L.O. and Y.Z. who performed all their own sampling, laboratory incubations and gas chromatography data collection. Blinding in this situation was therefore not possible.

Did the study involve field work? ☒ Yes ☐ No

Field work, collection and transport

Field conditions

For sediment sample collection in 2013 and 2016, as the sediments were kept intact in their corers in a cool-box before incubation and no treatment was applied to samples in the field, no field conditions beside water temperature were recorded. Water samples collected in August 2020 with an average water temperature was 15°C.

Location

The 236 streams used to relate delivery of excess fine sediment to streambed organic matter are distributed throughout Wales and England and their positions are provided in Figure 1 of the main text. The smaller number of streams where sediment and water samples were collected were in the south of England and their positions are provided in Supplementary Figure 1.

Access & import/export

The majority of the sampling sites were open to the public; otherwise the approval was given by the landlord before accessing.

Disturbance

The wider survey of fine sediment on 236 streambeds used - by definition - the disturbance technique, but the 4 samples (0.1m^2) collected would have only disturbed ~2% of the typical 3.5m x 5m reach of stream and any evidence of this was rapidly removed by stream flow. For the incubations, the collected cores of sediment were <80cm^2 and any disturbance to the wider streambed was therefore minimal.

Reporting for specific materials, systems and methods

We require information from authors about some types of materials, experimental systems and methods used in many studies. Here, indicate whether each material, system or method listed is relevant to your study. If you are not sure if a list item applies to your research, read the appropriate section before selecting a response.

Materials & experimental systems

n/a	Involved in the study	
☒	☐	Antibodies
☒	☐	Eukaryotic cell lines
☒	☐	Palaeontology and archaeology
☒	☐	Animals and other organisms
☒	☐	Human research participants
☒	☐	Clinical data
☒	☐	Dual use research of concern

Methods

n/a	Involved in the study	
☒	☐	ChIP-seq
☒	☐	Flow cytometry
☒	☐	MRI-based neuroimaging
